# C-reactive protein, *APOE* genotype and longitudinal cognitive change in an older population

**DOI:** 10.1093/ageing/aft193

**Published:** 2013-12-03

**Authors:** Thomas A. S. Lima, Amanda L. Adler, Thais Minett, Fiona E. Matthews, Carol Brayne, Riccardo E. Marioni, on behalf of the Medical Research Council Cognitive Function and Ageing Study

**Affiliations:** 1 Hospital Militar de Área de Brasília, Brasília, Brazil; 2 Wolfson Diabetes and Endocrine Clinic, Institute of Metabolic Sciences, Addenbrooke's Hospital, Cambridge CB2 2QQ, UK; 3 Department of Public Health and Primary Care, University of Cambridge, Cambridge CB2 0SR, UK; 4 MRC Biostatistics Unit,Cambridge CB2 0SR, UK; 5 Department of Psychology, University of Edinburgh, 7 George Square, Edinburgh, EH8 9JZ, UK; 6 Centre for Cognitive Ageing and Cognitive Epidemiology, University of Edinburgh, 7 George Square, Edinburgh, EH8 9JZ, UK

**Keywords:** C-reactive protein, *APOE*, cognitive decline, older population, older people

## Abstract

**Background:** circulating measures of inflammatory markers, such as C-reactive protein (CRP) have been associated with an increased risk of future cognitive decline. However, the nature of the relationship among the very old (>75 years) is unclear. Cross-sectional evidence suggests that elevated CRP may even be protective in this age group. This study examines these associations longitudinally.

**Methods:** logistic regression was used to investigate the association between CRP and drop in cognitive performance (≥3 point change on the Mini-Mental State Examination) over a 4-year period in a population of 266 people, mean age 77 years.

**Results:** increased levels of CRP were associated with a decreased risk of a drop in cognitive performance; however, this association was only seen in those without an *APOE* e4 allele [odds ratio of decline per unit increase in ln(CRP) 0.57, *P* = 0.04]. The magnitude of the finding remained consistent after adjustment for cardiovascular confounders (smoking, drinking, MI, stroke, diabetes, education, medication and blood pressure). For those with an e4 allele, the relationship with longitudinal cognitive decline was neither statistically significant nor in a consistent direction after controlling for acute inflammation.

**Conclusions:** this study strengthens previous cross-sectional findings and shows elevated levels of CRP to be linked to a decreased risk of longitudinal cognitive decline in the very old. However, as with prior analyses, this was only observed in those not carrying an *APOE* e4 allele. Future work on larger *APOE* e4 allele carrying samples is required to determine the nature of the association in this population.

## Introduction

Circulating serum and plasma levels of the inflammatory marker C-reactive protein (CRP) have been associated with cognitive decline and risk of dementia in ageing populations [1–3]. However, recent studies have questioned the nature of this relationship in the very old (>75 years). For example, Silverman *et al.* [4] found that increased concentrations of CRP were linked cross-sectionally to improved memory performance in later life, but only in subjects without an *APOE* (apolipoprotein) e4 allele. However, null findings were observed for a global measure of cognition (Mini-Mental State Examination, MMSE) and for measures of language/executive function. Moreover, in a separate study [5], Silverman *et al*. also showed that parents and siblings of older adults with high CRP but no cognitive impairment, had a reduced risk of dementia compared with relatives with low CRP and no cognitive impairment. Associations between raised levels of other cardiovascular risk factors and more favourable cognitive trajectories have also been observed for body mass index and blood pressure [6].

The aim of this paper was to investigate the relationship between late-life CRP, its interaction with *APOE* e4 and drop in longitudinal cognitive performance in a population-based sample of older subjects.

## Methods

### Population

The analysis cohort is a subset of the Medical Research Council Cognitive Function and Ageing Study (CFAS). The population frame comprised 13,004 individuals aged 65 years or over living in five geographic areas of the UK, who were selected from local Family Health Service Authority lists. The response rate was 81% and data collection began in the early 1990s [7]. The specific subset analysed in this study comprises 1,733 participants [mean age 80.0 (SD 6.8), 64% female] with the mean follow-up of 4.1 years between years 1997 and 2001 (6- and 10-year follow-up, respectively). We analysed data from 273 participants who had data on both serum CRP concentrations and MMSE, and who were free from dementia at the 6-year ‘baseline’. CFAS has multi-centre research ethics committee's approval and ethical approval from the relevant local research ethics committees.

### Exposure and covariates

CRP was the main exposure of interest and was measured on a Dade-Behring Dimension analyser by the Nutritional Biolanalysis Laboratory, MRC Human Nutrition Research Unit, Elsie Widdowson Laboratory, Cambridge. Information on age, gender, average weekly intake of alcohol, *APOE* genotype, education, regular medication use and cardiovascular risk factors, such as a history of stroke, myocardial infarction requiring medical attention and smoking status, were available for most individuals. These variables were assessed once at the baseline wave. Genotyping for *APOE* was carried out in line with Wenham *et al.* [8].

### Outcome

Cognitive function was assessed by the MMSE [9], which has a discrete value range between 0 and 30 points. Cognitive decline was defined as a decrease of 3-or-more points over the 4-year follow-up period (1997–2001).

### Statistical analysis

Logistic regression was used to test the association between log-transformed CRP and cognitive decline. Prior to performing the analysis, we excluded individuals who had a baseline MMSE score <18, which is compatible with severe cognitive impairment (*n* = 2). We also trimmed the CRP data for outliers that were >3 SD beyond the mean (*n* = 5). This left an analysis sample of 266 subjects. An initial model adjusted for age and sex, followed by models stratifying by *APOE* status (e4 carrier or not). A saturated model was also built to allow for adjustment for potential confounders. Owing to a lack of observations in the *APOE* e4 carriers subpopulation, this step was only performed for those without an e4 allele. Analyses were performed using the statistical software ‘R’.

## Results

Data from 266 participants (56% female), mean age 77 (SD 5.4, range 69.6 to 96.5) years at baseline were analysed (Table 1). The majority of the population were non-smokers (80%) with low-to-moderate alcohol consumption (65%). Approximately 41% of subjects had high blood pressure, 13% (4%) had a history of myocardial infarction (stroke), 8% had diabetes mellitus and almost all were on regular medication. Over a mean of 4.1 years of follow-up, 86 participants developed cognitive decline (64% females).

**Table1.** Descriptive statistics at baseline: maximum *n* = 266

	Median	IQR
Serum CRP (mg/l)	3.8	(2.6, 6.0)
	Mean	SD
Age at baseline (years)	77.1	5.5
Education (years)	9.6	1.7
	*n*	%
Sex (F)	148	55.6
*APOE* e4 carrier (*n* = 258)	54	20.9
Alcohol (normal/moderate) (*n* = 232)	150	64.7
Alcohol (severe)	52	22.4
Smoking (current/ex)	53	19.9
Medication (*n* = 264)	243	92.0
Diabetes mellitus	20	7.5
Myocardial infarction	35	13.2
High blood pressure	110	41.4
Stroke	11	4.1

Age- and sex-adjusted odds ratios for log(CRP) in relation to cognitive decline are displayed in Table 2. For every unit increase in log(CRP), there was an 21% decrease in the risk of decline in cognition (OR: 0.79, 95% CI: 0.51–1.20). However, this association was not statistically significant (*P* = 0.28). In accordance with the analyses by Silverman *et al.*, we stratified by the *APOE* e4 status. In the group without an e4 allele, log(CRP) was associated with a decreased risk of longitudinal cognitive decline (OR: 0.57, 95% CI: 0.33–0.96, *P* = 0.04). In the group carrying an e4 allele, log(CRP) was not significantly associated with cognitive decline, although the point estimate for the relationship was in the opposite direction from those without an e4 allele (OR: 1.54, 95% CI: 0.65–3.79, *P* = 0.32). Sensitivity analyses excluding those with a CRP level >10 mg/l (acute inflammation) resulted in minor changes to the magnitude of the associations for those without an e4 allele (OR: 0.54, 95% CI: 0.26–1.12, *P* = 0.10). For those with an e4 allele, the association remained non-significant but the point estimate changed direction (OR: 0.72, 95% CI: 0.15–3.04, *P* = 0.65). A final model was built to covary for potential confounders in those without an e4 allele; there were too few observations to do this for the e4 allele subgroup. After adjusting for diabetes, education, medication, alcohol consumption, blood pressure, stroke, smoking and MI, the strength of the association between log(CRP) and cognitive decline remained similar to the crude age- and sex-adjusted model (OR: 0.57, 95% CI: 0.30–1.04, *P* = 0.08).

**Table 2.** Logistic regression analyses of CRP on cognitive decline (3 or more point drop on the MMSE)

	Basic model (*n* = 266)				*APOE* e4 non-carriers (*n* = 204)				*APOE* e4 carriers (*n* = 54)			
	OR^a^	95% CI		*P*-value	OR	95% CI		*P*	OR		95% CI	*P*
Intercept	2.6 × 10^−4^	4.9 × 10^−6^	0.012	<0.001	3.1 × 10^−5^	2.3 × 10^−7^	0.003	<0.001	0.06	1.1 × 10^−5^	231.44	0.50
log(serum CRP)	0.79	0.51	1.20	0.28	0.57	0.33	0.96	0.04	1.54	0.65	3.79	0.32
Age at baseline (years)	1.10	1.05	1.16	<0.001	1.14	1.08	1.22	<0.001	1.01	0.91	1.13	0.78
Sex (F)	1.38	0.80	2.41	0.25	1.00	0.52	1.92	0.99	3.22	0.97	12.04	0.06

^a^Odds ratio.

## Discussion

In this prospective study, we found increased concentrations of serum CRP to be associated with a decreased risk of drop in cognitive performance (measured as a decline of 3 or more points on the MMSE over a 4-year period) in the very old. However, this association was only seen in those without an *APOE* e4 allele. The magnitude of the finding remained consistent after comprehensive adjustment for potential confounders. For those with an e4 allele, the relationship with cognitive decline was neither statistically significant nor in a consistent direction after controlling for acute inflammation.

These findings are broadly in line with recent papers [4, 5, 10] that describe an association between raised CRP and better cognitive outcomes in the very old. One study also found the associations to only be present in those without an *APOE* e4 allele [4]. However, different from our study, Silverman *et al.* [4] found a link with a measure of memory, but not general cognition (MMSE scores). One possible explanation for these differences is the cross-sectional design of their study.

A strength of our study is its prospective design which enabled us to evaluate differences in cognition (MMSE) over time. Limitations include only one measurement of CRP, which could contribute to regression dilution bias. Survival bias may have occurred since those with lower cognitive scores are more likely to die. A further limitation is the small subpopulation of *APOE* e4 allele carriers. The number and proportion of carriers were similar to that from Silverman's study [4] [*n* = 50 (29%) versus *n* = 54 (21%)]. Much larger studies are needed to investigate the direction and magnitude of this association.

This study draws attention to the concept of reverse epidemiology, where traditional risk factors may actually be protective within a population subset. Silverman *et al.* [5] hypothesised that an active immune system in later life may be important in protecting against dementia. The findings from our analysis imply that this hypothesis may also be relevant in non-pathological cognitive ageing.

In conclusion, while elevated levels of CRP in mid- and early late-life predict subsequent cognitive impairment and dementia, this role appears to be reversed in the very old. However, this association was only observed in older people who do not carry an *APOE* e4 allele. Nonetheless, the magnitude of the association is not influenced by cardiovascular disease, and lifestyle factors such as smoking and drinking.

**Key points**

- Cross-sectional evidence suggests that elevated CRP in late-life is linked to better cognition.
- We show, longitudinally, that raised CRP is linked to a lower risk of cognitive decline in those without an *APOE* e4 allele.
- In the very old, the relationship between CRP and cognitive decline in those carrying an *APOE* e4 allele is unclear.

## Conflicts of interest

None declared.

## Funding

This work was supported by a Medical Research Council (MRC) project grant (MRC G9901400). F.E.M. is funded under MRC programme grant UC_US_A030_0031. R.E.M. is an Alzheimer's Research UK Fellow (ART-RF2010-2) and a member of the University of Edinburgh Centre for Cognitive Ageing and Cognitive Epidemiology, part of the cross council Lifelong Health and Wellbeing Initiative (G0700704/84698), for which funding from the Biotechnology and Biological Sciences Research Council, Engineering and Physical Sciences Research Council, Economic and Social Research Council is gratefully acknowledged.
